# Make Sure You Have a Safety Net: Updates in the Prevention and Management of Infectious Complications in Stem Cell Transplant Recipients

**DOI:** 10.3390/jcm9030865

**Published:** 2020-03-21

**Authors:** Carlos A. Q. Santos, Yoona Rhee, Michael T. Czapka, Aamir S. Kazi, Laurie A. Proia

**Affiliations:** Rush University Medical Center, 600 S. Paulina St., Suite 143, Chicago, IL 60612, USA; Yoona_Rhee@rush.edu (Y.R.); Michael_T_Czapka@rush.edu (M.T.C.); Aamir_S_Kazi@rush.edu (A.S.K.); Laurie_Proia@rush.edu (L.A.P.)

**Keywords:** infectious complications, cytomegalovirus, invasive fungal infections, antibacterial prophylaxis, antibacterial treatment

## Abstract

Hematopoietic stem cell transplant recipients are at increased risk of infection and immune dysregulation due to reception of cytotoxic chemotherapy; development of graft versus host disease, which necessitates treatment with immunosuppressive medications; and placement of invasive catheters. The prevention and management of infections in these vulnerable hosts is of utmost importance and a key “safety net” in stem cell transplantation. In this review, we provide updates on the prevention and management of CMV infection; invasive fungal infections; bacterial infections; *Clostridium difficile* infection; and EBV, HHV-6, adenovirus and BK infections. We discuss novel drugs, such as letermovir, isavuconazole, meropenem-vaborbactam and bezlotoxumab; weigh the pros and cons of using fluoroquinolone prophylaxis during neutropenia after stem cell transplantation; and provide updates on important viral infections after hematopoietic stem cell transplant (HSCT). Optimizing the prevention and management of infectious diseases by using the best available evidence will contribute to better outcomes for stem cell transplant recipients, and provide the best possible “safety net” for these immunocompromised hosts.

## 1. Introduction

Hematopoietic stem cell transplant (HSCT) recipients are at increased risk of infection and immune dysregulation due to reception of cytotoxic chemotherapy regimens (which may induce neutropenia, hypogammaglobulinemia, lymphopenia and mucosal barrier injury), development of graft versus host disease (GVHD) (thereby warranting treatment with immunosuppressive medications) and the presence of invasive catheters [1]. The prevention and management of infections in these vulnerable hosts is important, and a key “safety net” in HSCT.

## 2. Cytomegalovirus

### 2.1. Letermovir—A Novel Drug for Cytomegalovirus Prophylaxis

Cytomegalovirus (CMV) reactivation can be harmful to HSCT recipients and is associated with increased mortality, despite currently available antivirals [2]. Given the severity of illness from CMV end-organ infection, various CMV monitoring and treatment strategies are used in the HSCT population to prevent viremia and end organ disease [3]. A commonly used pre-emptive strategy after HSCT consists of interval monitoring for CMV replication with quantitative CMV nucleic acid testing after transplant, followed by the administration of antiviral agents such as ganciclovir, valganciclovir or foscarnet upon the detection of subclinical CMV viremia. However, these medications have toxicities which can limit their use in HSCT recipients.

In 2017, a novel antiviral agent, letermovir, was approved for CMV primary prophylaxis (as opposed to pre-emptive therapy) in allogeneic HSCT recipients older than 18 years old who are known to be CMV-serology positive. Letermovir inhibits the UL56 component of the viral terminase complex which is responsible for cleaving DNA concatemers for insertion into viral capsids [4]. The terminase complex is composed of viral proteins UL56, UL89 and UL51. A phase III, double-blinded, randomized controlled trial was performed comparing the effectiveness of this new antiviral to placebo in preventing CMV disease and mortality [5]. CMV-seropositive patients over 18 years of age receiving allogenic HSCT (R+) without active viremia were enrolled. Participants were randomized to either approximately 100 days of letermovir (480 mg daily versus 240 mg if the patient was taking a calcineurin inhibitor) post-HSCT, or placebo, a median of nine days following transplantation. Patients underwent weekly and subsequent biweekly surveillance for CMV viremia, with the primary endpoint being clinically significant CMV infection by post-transplant week 24, defined as either starting pre-emptive therapy with previously approved agents or having evidence of end-organ disease. Patients receiving letermovir had a 37.5% incidence of the primary endpoint compared to 60.6% in the placebo group (*p* < 0.001). All-cause mortality at 24 weeks post-transplant occurred in 10.2% of patients in the treatment group versus 15.9% of patients who received placebo (*p* = 0.03). There was no difference in mortality at 48 weeks post-transplant. Secondary effects of letermovir included nausea, atrial tachyarrhythmias and edema; however, none of the adverse events approached a statistically significant threshold when compared to placebo-associated reactions. Time to engraftment was not significantly different in the two arms. The trial described a patient who developed a UL56 (terminase component) mutation during the study which conferred letermovir resistance. At least one other HSCT patient on letermovir prophylaxis has been described with a UL56 mutation following approval [6].

While the letermovir study validates its effectiveness in preventing CMV viremia and end-organ disease using a primary prophylaxis approach, questions remain as to how this would compare with the commonly used pre-emptive treatment strategy in real-world settings [3], and what post-marketing reports will reveal regarding side-effects with expanded use and longer durations of treatment.

Both intravenous (IV) and oral formulations of letermovir are available, and dosing is suggested at 480 mg daily or 240 mg daily if cyclosporine is co-administered [5]. Letermovir has not been studied in patients with creatinine clearance (CrCl) of less than 10 mL/min, dialysis patients or severe liver impairment defined as Child–Pugh class C. As letermovir targets the CMV-specific terminase complex, it does not demonstrate activity against other herpesviruses, and prophylaxis against HSV should be given if warranted. Its target is distinct from DNA polymerase, which is targeted by ganciclovir, cidofovir and foscarnet, and mutations that render these agents ineffective (UL97 and UL54) would not confer resistance to letermovir [7]. Letermovir has important interactions with commonly used immunosuppressive drugs due to effects on cytochrome P4503A and organic anion transporters. Letermovir decreases voriconazole levels by inducing CYP2C9/19, but does not alter posaconazole levels [8]. Coadministration of letermovir with cyclosporine, tacrolimus and sirolimus leads to increased exposure to these immunosuppressive drugs; therefore, a dose of 240 mg daily is suggested if cyclosporine is co-administered [9]. In contrast, letermovir does not alter exposure to mycophenolate. Additionally, letermovir can increase drug concentrations of amiodarone, glyburide, repaglinide, rosiglitazone and HMG-CoA reductase inhibitors, such as atorvastatin, simvastatin and pravastatin; and decrease drug concentrations of warfarin, phenytoin, omeprazole and pantoprazole. More drug interactions are likely to be identified with increased use of letermovir. Drug interaction checks and comprehensive medication management are essential prior to starting letermovir.

Despite the pitfalls with medication interactions and the possibility of developing viral resistance, the secondary effect profile for letermovir appears relatively benign, and presents an advance in CMV prophylaxis by avoiding the myelosuppression and renal toxicity of typically used CMV-active drugs. More research is needed to determine whether all HSCT recipients should be receiving primary prophylaxis with letermovir as opposed to the widely-used pre-emptive treatment approach, or whether restricting letermovir to those individuals at highest risk of CMV infection would be more cost-effective. There has been interest in using this medication off-label for treatment of drug-resistant CMV infection, but no clinical trials have yet addressed this indication.

#### Key Points

- Letermovir, a UL56 viral terminase complex inhibitor, can be used by CMV-seropositive HSCT recipients to prevent CMV viremia and disease.
- Unlike valganciclovir and ganciclovir, letermovir has no activity against HSV; acyclovir prophylaxis to prevent HSV should be administered in addition to letermovir if indicated.
- Letermovir does not cause myelosuppression, which offers an advantage over valganciclovir and ganciclovir.
- Letermovir has many drug interactions due to its effects on cytochrome P4503A and organic anion transporters. Drug interaction checks and comprehensive medication management are essential prior to starting letermovir.
- Letermovir may have a lower barrier to resistance compared to valganciclovir or ganciclovir, and breakthrough CMV infections on letermovir prophylaxis can occur.

### 2.2. New Therapeutics in Resistant Cytomegalovirus Infection

CMV infections are typically managed with ganciclovir or valganciclovir and with reduction of immunosuppression when able. CMV infections resistant to ganciclovir, or patients intolerant of ganciclovir, are often treated with foscarnet, or less commonly, cidofovir. Foscarnet and cidofovir are limited by nephrotoxicity. CMV mutations have emerged that confer resistance to all typically utilized antivirals, and HSCT recipients are at particular risk given their immunocompromise in the setting of GVHD [10]. Given the high morbidity and mortality of CMV with end-organ involvement in the HSCT population [2], and the problems of drug-resistance and toxicities, novel therapeutic approaches are being utilized. We describe recent data on letermovir, leflunomide and CMV-specific adoptive T-cell transfer.

#### 2.2.1. Letermovir

Letermovir, a novel antiviral agent with activity solely against CMV, has been approved for use in primary prophylaxis of CMV. The rationale for utilizing letermovir in drug-resistant CMV is that viral terminase, the molecular target, is unrelated to viral DNA replication and resistance generating mutations seen with typical therapies [7]. There are no randomized trials utilizing this medication for treatment, but there are case reports of varying success describing the use of letermovir for CMV infection. A case series of patients with CMV retinitis resistant to conventional therapies demonstrated symptomatic improvement in retinitis with letermovir, in combination with CMV hyperimmune immune globulin (CMV IG) and intravitreal foscarnet or ganciclovir [11]. However, persistent CMV viremia and concerns for acquired letermovir resistance eventually necessitated alternative therapies in a majority of the patients. A lung transplant recipient has also been reported with multi-organ involvement of CMV; the patient failed treatment with ganciclovir, foscarnet, cidofovir, leflunomide, CMV IG, brincidofovir and an artemisinin derivative, but eventually had clinical improvement and sustained viral suppression with letermovir [12].

#### 2.2.2. Leflunomide

While typically used in the treatment of rheumatologic disorders, leflunomide has been used for drug-resistant CMV [13]. In-vitro efficacy has been described against resistant CMV with inhibition of tegument acquisition by viral capsids [14]. There are no randomized clinical trials for leflunomide treatment of CMV. A recent case series of lung transplant recipients with CMV infection described treatment and secondary prophylaxis with leflunomide [15]. Most of the transplant recipients had drug resistant virus and had received unsuccessful prior therapy with other agents, including ganciclovir, valganciclovir, CMV-specific IVIG or foscarnet; however, 77.7% had an initial decrease and 58.3% had long-term suppression of viremia after starting leflunomide [15]. Leflunomide is loaded and followed by maintenance dosing. It carries a black box warning for hepatotoxicity, and monitoring for this effect as well as myelosuppression, nephrotoxicity and peripheral neuropathy are suggested [13].

#### 2.2.3. CMV-Specific T-Lymphocytes

Another agent under investigation for treatment of CMV infection in HSCT recipients is CMV-specific T-lymphocytes. In HSCT, the conditioning regimen typically results in lymphopenia and depletion of CD8 CMV-specific lymphocytes, which leads to increased risk of CMV infection or reactivation [16]. A small phase I trial was conducted on HSCT recipients who had CMV positive donors (recipients had mixed CMV serologic status) and received preventative CMV-specific CD8 cells in escalating dosages starting 30 days post-transplant [17]. No adverse events from the infusions were reported, CMV viremia was not detected and a dose-response trend for CMV cytotoxic activity was apparent. A subsequent phase II single arm trial was conducted in patients receiving allogenic HSCT from CMV positive donors, and HSCT recipients received CMV-specific CD8 and CD4 T-cells approximately 28 days following transplant [18]. In this trial, the incidence of GVHD in patients who received CMV T-cells was comparable to the expected incidence of allogenic HSCT recipients overall, and no other significant adverse events were reported with cell infusions. There was evidence of replication of donor-CMV-specific T-lymphocytes in recipients and maintenance of CMV cytotoxic activity over time. The absence of delayed-onset CMV infection in the population was lower than for the general allogenic HSCT population. Given the small size of the study and patient heterogeneity, it is difficult to draw definitive conclusions regarding efficacy of this therapy. These studies, however, show good preliminary data that can be used to inform more clinical investigations.

Reports on the clinical success of CMV-specific T-lymphocytes have been variable. A 2016 case report describes a 35 year old patient with acute myeloid leukemia who underwent allogeneic HSCT from a mismatched unrelated donor (CMV R+/D-) that was complicated by GVHD and CMV colitis [19]. The patient failed therapy with ganciclovir, CMV-IG, foscarnet, cidofovir and brincidofovir, and developed significant medication-related toxicities. Given UL97 and UL54 mutations, drug toxicity and the absence of a CMV-specific immune response as determined by QuantiFERON CMV assay [20], CMV-specific CD8 T-cells were sourced from the patient’s sibling and administered without demonstrable effect. Unfortunately, the patient succumbed to CMV colitis and GVHD. By comparison, a small case series of HSCT recipients, who experienced treatment failure after a median of eight weeks of conventional CMV therapy for viremia, experienced clearance of viremia (the majority of patients at least) following transfer of CMV-specific CD4 lymphocytes [21]. This study also found longer persistence of investigational cell lines with CD4 cells, as well as expansion of CMV-specific CD8 T cells through adoptive transfer of CD4 lymphocytes. In another case report, a 50 year old man with follicular lymphoma (CMV R+/D-) who received a matched unrelated allogenic HSCT complicated by GVHD incurred UL97 mutations on valganciclovir treatment during the fourth recurrence of CMV [22]. Cidofovir, foscarnet and leflunomide were administered, which failed to control viremia. The patient received a second HSCT while viremic, but subsequently developed retinitis on foscarnet therapy with subsequent acquisition of UL54 mutations. He was given maribavir and artesunate, which decreased CMV viremia. Mixed CD3 lymphocytes specific for CMV were then infused and after one month his CMV viral load was found to be undetectable, and his retinitis eventually improved. It is unclear whether the clinical response was due to the maribavir plus artesunate combination or from T-cell infusion.

As demonstrated above, clinical efficacy and preparations of CMV-specific T-lymphocytes vary. More studies are needed. However, immunotherapy can be considered in patients with refractory and resistant CMV infections, or for HSCT recipients who cannot tolerate conventional therapy.

##### Key Points

- Letermovir is an inhibitor of viral terminase that can be used to treat CMV resistance to conventional therapy with ganciclovir, foscarnet or cidofovir; it has a lower barrier to resistance, but has been used in combination with other antiviral agents.
- Leflunomide, a drug used in the treatment of rheumatologic disorders, can inhibit tegument acquisition by viral capsids, and has been used for patients with drug-resistant CMV; it carries a black box warning for hepatotoxicity, and can also cause myelosuppression, nephrotoxicity and peripheral neuropathy.
- CMV-specific T-lymphocytes have been used to treat refractory and resistant CMV disease; clinical efficacy and preparations of CMV-specific T-lymphocytes vary.

## 3. Invasive Fungal Infections

### 3.1. Advances in Antifungal Prophylaxis

Patients undergoing HSCT are at increased risk of invasive fungal infections (IFIs) due to neutropenia during the pre-engraftment period or from severe GVHD in the post engraftment phase [23]. Following HSCT, *Candida* accounts for the majority of invasive yeast infections and *Aspergillus* accounts for most invasive mold infections [24].

#### 3.1.1. Antifungal Prophylaxis for HSCT

In a national prospective surveillance study, the overall incidence of IFIs was 3.4% in HSCT recipients [24]. *Aspergillus* is the most commonly encountered IFI in this patient population, followed by candidiasis. The incidence of IFIs is lower for autologous HSCT than allogeneic HSCT recipients.

Antifungal prophylaxis is recommended for HSCT patients expected to have profound (absolute neutrophil count <100/µL) and protracted (neutropenia lasting ≥7 days) neutropenia with accompanying mucositis [23]. Of the triazole antifungals, fluconazole has activity against yeast but not mold, whereas posaconazole, voriconazole and isavuconazole have activity against yeast and mold.

When voriconazole and fluconazole were compared for IFI prophylaxis after HSCT, a trend towards fewer IFIs (7.3% vs. 11.2%; *p* = 0.12), a lower number of *Aspergillus* infections (9 vs. 17; *p* = 0.09) and lesser empirical antifungal use (24.1% vs. 30.2%; *p* = 0.11) were observed at 6 months with voriconazole [25]. However, no difference was seen in the primary endpoints of fungal-free survival (75% vs. 78%; *p* = 0.49). Similarly, no significant difference was observed for the secondary endpoints of overall survival and drug toxicities at 6 and 12 months. Patients enrolled in this study were deemed to be at lower risk for IFI and mortality because of lower rates of GVHD and enrollment of fewer patients with underlying acute myeloid leukemia (AML). The lack of benefit from voriconazole in this study may be attributed to inadequate bioavailability, since voriconazole levels were not monitored. A subgroup analysis of patients with AML who underwent HSCT showed fewer IFIs in the voriconazole group (8.5% vs. 21%; *p* = 0.04) and greater fungal-free survival (78% vs. 61%; *p* = 0.04), but no difference in overall survival (81% vs. 72%, *p* = 0.32).

A comparison of voriconazole and itraconazole following allogeneic HSCT revealed equivalent results for IFI prevention at 180 days (1.3% vs. 2.1%, *p* = 0.54) and overall survival at one year (73.5% vs. 67.0%, *p* = 0.17) [26]. Voriconazole was better tolerated at 100 days of prophylaxis with less interruptions in therapy (53.6% vs. 39.0%, *p* < 0.01). Gastrointestinal disturbances were the predominant treatment-limiting adverse effects of itraconazole, whereas hepatotoxicity and visual impairment were common with voriconazole.

In a randomized multicenter study, posaconazole was superior to either fluconazole or itraconazole in preventing proven or probable IFIs in neutropenic patients who received chemotherapy for AML or myelodysplastic syndrome (8% vs. 2%; *p* < 0.001) [27]. This difference was noted after 12 weeks of prophylaxis, until occurrence of an IFI or recovery from neutropenia with complete remission. Moreover, better survival was noted in the posaconazole group (84% vs. 78%; *p* = 0.048). Adverse events attributable to the treatment were higher in the posaconazole group, with cardiac-related adverse events occurring only with posaconazole. Although this study did not assess HSCT recipients, this trial highlights the role of posaconazole as a prophylactic agent and warrants further research in patients undergoing HSCT. Notably, higher serum concentrations are achieved with posaconazole delayed-release tablets in comparison to the suspension formulation, which requires food and preferably a high fat meal.

Isavuconazole is a newer mold-active azole that has been utilized for primary prophylaxis, although it has not been studied for prophylaxis in randomized controlled trials. Higher rates of breakthrough invasive fungal infections (bIFIs) have been observed with isavuconazole prophylaxis (10.2%) in comparison to posaconazole (4.1%) or voriconazole (1.1%) in a retrospective analysis of denovo or relapsed/refractory AML patients [28]. Of the 12 total bIFIs, 11 occurred in AML or (acute lymphoblastic leukemia) ALL patients receiving chemotherapy, and one occurred in an HSCT recipient. Incidence of invasive pulmonary aspergillosis was also notably higher for isavuconazole (6.8%) compared to posaconazole (1.3%) and voriconazole (0%). Additional reports of bIFIs while on isavuconazole prophylaxis in patients with hematologic malignancies underscores the need for further research on the role of isavuconazole for primary fungal prophylaxis [29].

The National Comprehensive Cancer Network (NCCN) guidelines continue to recommend fluconazole as the azole of choice for neutropenic, allogeneic HSCT recipients [30]. Given the information reviewed above however, the panel recommends posaconazole and voriconazole as alternative options. Antifungal prophylaxis is recommended for the duration of neutropenia and for at least 75 days after allogeneic HSCT. Antifungal prophylaxis with fluconazole is recommended for autologous HSCT recipients with mucositis, whereas no fungal prophylaxis is recommended in the absence of mucositis.

#### 3.1.2. Antifungal Prophylaxis for GVHD

A study comparing posaconazole and fluconazole for IFI prophylaxis in GVHD patients remains the only randomized, blinded trial in this subset of patients. Enrolled patients were HSCT recipients with acute GVHD, grade II–IV GVHD or chronic extensive GVHD; or recipients of intensive immunosuppressive therapy consisting of high-dose corticosteroids, antithymocyte globulin or an immunosuppressive regimen combining two or more agents [31]. After nearly a four-month treatment period, posaconazole was equivalent to fluconazole in preventing all IFIs (incidence, 5.3% and 9.0%, respectively; *p* = 0.07) but was superior to fluconazole in preventing invasive aspergillosis (2.3% vs. 7.0%; *p* = 0.006). Breakthrough IFIs were notably lesser in the posaconazole group (2.4% vs. 7.6%, *p* = 0.004), including bIFIs from invasive aspergillosis. Although both groups had similar overall mortality, fewer deaths from IFIs were observed in the posaconazole group (1%, vs. 4%; *p* = 0.046). Treatment-related adverse events were found to be similar in both groups.

The NCCN guidelines recommend fungal prophylaxis with posaconazole for all GVHD patients receiving immunosuppressive therapy, especially patients receiving intensive immunosuppressive therapy [30]. The benefit from posaconazole prophylaxis has not yet been established for GVHD patients receiving less intensive immunosuppressive therapy. Voriconazole may be considered an alternative for mold-active prophylaxis. Duration of prophylaxis is recommended until resolution of significant GVHD.

##### Key Points

- Antifungal prophylaxis is recommended for HSCT patients expected to have profound (<100/µL) and protracted (≥7 days) neutropenia with accompanying mucositis, and allogeneic HSCT recipients with GVHD.
- Fluconazole remains the antifungal of choice for HSCT recipients; voriconazole and posaconazole can be used for higher-risk patients.
- A comparison of voriconazole and fluconazole for IFI prophylaxis after HSCT showed a trend towards fewer IFIs in the voriconazole arm, but equivalent overall survival.
- A comparison of posaconazole and fluconazole or itraconazole for IFI prophylaxis in neutropenic patients with AML or (myelodysplastic syndrome) MDS showed fewer IFIs and greater survival in the posaconazole arm.
- A comparison of posaconazole and fluconazole for IFI prophylaxis in patients with GVHD showed lesser IFIs in the posaconazole arm, but similar overall mortality.

### 3.2. Treatment of Invasive Fungal Infections in HSCT

Despite advances in fungal diagnostics and the availability of new broad-spectrum antifungal therapies, mortality associated with IFI in HSCT remains high. Delayed engraftment with prolonged neutropenia and development of GVHD after allogeneic transplant continue to be major risk factors associated with IFI. While the widespread use of fluconazole prophylaxis has led to a decreased incidence in *Candida albicans* infections, this has been offset by a rise in non-albicans candidiasis and increased invasive mold infections, particularly due to *Aspergillus* species [24].

#### 3.2.1. Candidemia and Invasive Candidiasis

*Candida* is a normal commensal of human skin and mucosal surfaces of the gastrointestinal tract. *Candida* may cause systemic infection when there is a breach in skin or loss of mucosal integrity as a result of cytotoxic chemotherapy and gut mucositis. Autologous stem cell transplant recipients, along with mucositis and allogeneic stem cell transplant recipients, are at risk for developing invasive candidiasis, but routine use of azole prophylaxis has significantly reduced infection rates. The majority of *Candida* infections are caused by five main species: *C. albicans, C. glabrata, C. tropicalis, C. parapsilosis* and *C. krusei*. Although *C. albicans* is still the most common species identified clinically, the epidemiology of candidiasis has changed with an increase in the proportion of non-albicans species causing infection [32]. Development of candidiasis in the setting of neutropenia and prior fluconazole exposure may be caused by azole resistant species, such as *C. glabrata* and *C. krusei*, and outcomes are poor. Although there are no large, randomized controlled trials of treatment of candidemia in neutropenic patients, current guidelines recommend an echinocandin as first-line therapy for both neutropenic and non-neutropenic patients [33]. This recommendation is based on extrapolated data from studies in non-neutropenic patients showing the superiority of echinocandins as initial treatment for candidemia and invasive candidiasis [33,34,35]. The echinocandins, caspofungin, micafungin and anidulafungin, have a unique mechanism of action which is inhibition of glucan synthesis, and demonstrate in vitro activity against all species of *Candida,* including those that are azole resistant. The echinocandins are only available for intravenous administration but have minimal drug-drug interactions and an excellent safety profile owing to their specific effect on the fungal cell wall, which mammalian cells lack [36]. The echinocandins are considered fungicidal for *Candida,* although the emergence of echinocandin resistance, particularly with *C. glabrata*, is well described [37]. Lipid formulations of amphotericin B may be used as alternative therapy especially if echinocandin resistance is suspected or documented; however, the risk of nephrotoxicity, even with the lipid formulations, can limit their use. Fluconazole may be administered as step-down therapy to patients with candidemia or invasive candidiasis who have no significant prior azole exposure, are clinically stable and in whom the species of *Candida* causing infection is known. However, given the likelihood of previous azole exposure and the risk of azole resistance, fluconazole may be a more limited treatment option for patients with candidemia or invasive candidiasis following HSCT. While fluconazole does provide advantages—being nearly fully bioavailable; being well tolerated; and having excellent tissue penetration, including into sites such as the eye, brain and urine—in vitro susceptibility testing to ensure efficacy would be necessary. Of the newer azoles, only voriconazole has been approved as first-line treatment for candidemia and invasive candidiasis in non-neutropenic patients [38,39]. Voriconazole may not be reliable as initial therapy for neutropenic patients with candidemia owing to the likelihood of prior azole exposure and significant potential for cross resistance. Voriconazole could be given to select patients as continued oral therapy for *Candida* infections proven to be voriconazole susceptible, including those caused by non-albicans *Candida* species such as *C. glabrata* and *C. krusei*. Posaconazole is an extended spectrum azole approved for treatment of oro-esophageal candidiasis and fungal prophylaxis in patients with neutropenic AML/MDS and GVHD after HSCT but has not been studied for candidemia and is currently not recommended. Isavuconazole, the newest broad-spectrum azole, is FDA-approved for treatment of aspergillosis and mucormycosis, but in a large randomized controlled treatment trial of candidemia and candidiasis, isavuconazole was shown to be inferior to caspofungin [40].

The duration of treatment for uncomplicated candidemia is generally a minimum of 2 weeks after sterilization of the bloodstream; resolution of symptoms and signs of infection; and, ideally, neutrophil recovery. For patients who remain neutropenic, secondary azole prophylaxis may be warranted. There has been controversy regarding the need for catheter removal in neutropenic patients with candidemia. Removal of central venous catheters in non-neutropenic patients with candidemia is recommended; however, in neutropenic patients, additional considerations may be given to gut translocation as a source for candidemia and surgical risks associated with line removal [38]. The exception to this is candidemia due to *C. parapsilosis* which is nearly always associated with central venous catheter infection and for which line removal is necessary. Catheter retention must be weighed against the potential for *Candida* biofilm formation and the possibility of infection relapse if the line is maintained. A recent patient-level quantitative review of seven randomized candidemia treatment trials showed significantly improved survival associated with catheter removal during treatment, thereby leading some newer guidelines to recommend early catheter removal for all patients, including hematology patients [41,42]. *Candida* in the bloodstream can disseminate to virtually any organ, including the eye. All patients with candidemia should have a dilated funduscopic exam to look for endophthalmitis, since this can be a sight-threatening complication with a different management strategy if present; repeat eye exam after neutrophil recovery may be necessary, especially if visual symptoms are present. Chronic disseminated candidiasis, formerly known as hepatosplenic candidiasis, is a rare complication that may develop during neutropenia as a result of occult candidemia with hematogenous seeding of the liver or spleen, or in the setting of neutropenic mucositis with gut translocation of *Candida* into the portal venous system. In cases of disseminated candidiasis where fluconazole resistance is suspected or proven, broader antifungal treatment with an echinocandin or an extended spectrum azole such as voriconazole or posaconazole is given. Treatment for chronic disseminated candidiasis should be continued until there has been clinical and radiographic improvement along with neutrophil recovery. In patients with ongoing immunosuppression, secondary antifungal prophylaxis is recommended.

#### 3.2.2. Other Opportunistic Yeasts

Yeasts other than *Candida* may be seen rarely in patients following HSCT. Pulmonary or disseminated cryptococcal infection is uncommon in the setting of azole prophylaxis. Amphotericin B and the azoles, including fluconazole, have activity against *Cryptococcus*, whereas the echinocandins do not. Cryptococcal infection could arise in patients not receiving azole prophylaxis, or in patients with sub-therapeutic azole levels, as might be seen with itraconazole, voriconazole or posaconazole. Other rare yeasts reported to cause disseminated infection following HSCT include *Trichosporon, Saccharomyces, Rhodotorula, Geotrichum* and *Pseudozyma* [43]. These infections may manifest as breakthrough fungemia in patients with indwelling central venous catheters who are receiving antifungal prophylaxis. Antifungal susceptibility patterns can vary by isolate, although reduced susceptibility to echinocandins is common, and catheter removal is generally recommended [44,45].

#### 3.2.3. Invasive Molds

Opportunistic mold infections in HSCT recipients may occur following conditioning chemotherapy, particularly with delayed engraftment and prolonged neutropenia, or post-engraftment in those who develop GVHD. Of the opportunistic invasive molds, *Aspergillus species* cause the majority of infections. The primary portal of entry of *Aspergillus* is the upper or lower respiratory tract through inhalation of airborne spores. Pneumonia is the most common clinical manifestation of invasive aspergillosis, but sinusitis, orbital cellulitis, brain abscess and necrotizing skin infections can occur. Current treatment guidelines recommend voriconazole as first-line therapy for all forms of invasive *Aspergillus* infection [46,47]. This recommendation is based mainly on results of the Global *Aspergillus* Study, which was a multicenter, randomized controlled trial comparing voriconazole to conventional amphotericin B for primary treatment of proven or probable invasive aspergillosis; the majority of patients had an underlying hematologic malignancy or had undergone HSCT. Both clinical response and survival were significantly better in those patients who received voriconazole, and drug toxicity was significantly lower compared to amphotericin B [48]. Voriconazole is a broad-spectrum azole with in vitro and in vivo activity against most yeasts and molds. Voriconazole lacks activity against the *Mucorales* molds; breakthrough mucormycosis has been described in HSCT patients receiving long term voriconazole [49]. Voriconazole is available in a parenteral and oral formulation that has excellent bioavailability. Major limitations of voriconazole are cytochrome P450 drug-drug interactions, non-linear pharmacokinetics and inter-patient and intra-patient variability in metabolism, necessitating therapeutic drug monitoring to ensure that adequate serum levels are achieved [50,51]. Therapeutic drug monitoring is also warranted to ensure that voriconazole levels are not supra-therapeutic; high serum voriconazole concentrations may cause encephalopathy presenting as visual disturbances, hallucinations, delirium and confusion. For patients on chronic voriconazole, repeated sun exposure may cause photodermatitis and increased risk of squamous cell carcinoma, particularly in those on immunosuppression for GVHD [52]. Prolonged voriconazole use may also result in fluoride toxicity, owing to the presence of three fluorine groups on the molecule, manifesting as bone pain and periostitis that is reversible upon discontinuation of drug [53]. 

Alternative treatments for invasive aspergillosis include amphotericin B, the echinocandins, isavuconazole, posaconazole or itraconazole; fluconazole has no activity against *Aspergillus* and is not indicated for treatment. Amphotericin B is administered preferentially as a lipid formulation, the main advantage of which is the ability to administer higher doses with reduced nephrotoxicity compared to the conventional drug. However kidney injury may still occur in up to 20% of patients treated with a lipid formulation, especially in patients receiving concomitant nephrotoxic agents [54]. Amphotericin B lipid formulations do provide broader empiric coverage against *Aspergillus* and the agents of mucormycosis (e.g., *Rhizopus, Mucor*) for patients in whom an invasive mold infection is suspected but not yet proven. The exception to this is *Aspergillus terreus*, which can be amphotericin resistant, though infection with this species is uncommon.

Isavuconazole is the most recent extended spectrum azole to be approved for treatment of invasive aspergillosis and mucormycosis. Isavuconazole is available in both an IV and oral formulation as the prodrug, isavuconazonium sulfate, which is quickly broken down to active drug after administration; oral isavuconazole tablets have 98% bioavailability with absorption unaffected by food. Unlike voriconazole, isavuconazole exhibits dose-proportionate linear pharmacokinetics, has less potent cytochrome P450 inhibition and does not require routine therapeutic drug monitoring. Another advantage of isavuconazole, in contrast to other azoles, is that it does not cause QT prolongation [55]. Isavuconazole has broad in vitro activity against a variety of yeasts and molds, including moderate activity against the *Mucorales* molds. In a large, randomized, controlled, non-inferiority trial of isavuconazole versus voriconazole for treatment of *Aspergillus* and other invasive mold infections, there was no difference in clinical response or all-cause mortality between the two groups but fewer adverse events in the isavuconazole arm [56]. While isavuconazole does provide another treatment option for invasive aspergillosis, further long term studies are warranted, given recent reports of breakthrough IFIs, including potentially azole-resistant yeasts and molds, in high-risk patients receiving isavuconazole as either fungal prophylaxis or treatment [57].

While there are no prospective, comparative studies assessing the efficacy of posaconazole for first-line treatment of *Aspergillus*, given its broad-spectrum activity and proven benefit in preventing breakthrough aspergillosis in high-risk patients, posaconazole may be considered as alternative therapy. In an open label, multicenter retrospective study of posaconazole suspension as salvage therapy for patients with invasive aspergillosis, the overall success was 42% in the posaconazole-treated group and 26% in the control group [58]. Posaconazole was first released as an oral suspension with variable absorption requiring co-administration with food and an acidic beverage, and avoidance of concomitant acid-reducing medications; this posed challenges when administering posaconazole to patients with treatment-related stomatitis and anorexia, often resulting in sub-therapeutic serum levels [59]. Subsequently, posaconazole delayed-release (DR) tablets and IV posaconazole were approved for prevention of IFIs in high-risk patients [60]. Posaconazole DR tablets have improved absorption with less dependence on gastric acid compared to the suspension; this results in higher serum posaconazole concentrations without increase in hepatotoxicity [61,62]. Posaconazole does exhibit CYP3A4 mediated drug-drug interactions, though it is generally safe and lacks the CNS side-effects and photodermatitis seen with voriconazole. With the availability of the newer extended spectrum azoles, itraconazole is used less frequently for treatment of *Aspergillus* infections, but may still be considered as a second line of therapy in select patients with non-life-threatening infections who are intolerant of other antifungals.

Of the echinocandins, only caspofungin is approved for treatment of *Aspergillus* in patients who are refractory to, or intolerant of, standard therapy. This indication was based on a study of 83 patients, most with refractory infection, given caspofungin salvage therapy; a favorable response was observed in 45% [63]. Micafungin and anidulafungin have in vitro and clinical activity against *Aspergillus* and are presumed to be as effective as caspofungin given the relative similarity of each of the drugs in this class. However, there are no prospective, randomized controlled trials comparing an echinocandin to voriconazole for primary treatment of aspergillosis. Based on this, the echinocandins are not recommended as first-line monotherapy for *Aspergillus* infection, although they may have a role for use in combination antifungal regimens.

Given the persistently high mortality rates associated with invasive aspergillosis in high-risk patients, combination antifungal therapy has long been a treatment consideration, though strong evidence of its superiority over monotherapy is lacking. With the availability of several classes of antifungals with differing mechanisms of action, combination therapy may offer the potential for additive or synergistic benefits over monotherapy. The most compelling study of combination therapy comes from a large, prospective randomized trial comparing voriconazole monotherapy to voriconazole plus anidulafungin for treatment of invasive aspergillosis in patients with hematologic malignancies or HSCT [64]. There was a trend toward improved survival in the combination arm; six-week mortality was 19.3% in patients who received voriconazole plus anidulafungin versus 27.5% in patients given voriconazole alone. Subsequent analysis of 222 patients with probable invasive aspergillosis (defined as compatible radiographic findings with positive serum or broncho-alveolar lavage galactomannan antigen) found significantly lower mortality in the voriconazole plus anidulafungin group (16% versus 27%). While this study provides some suggestion that the combination of voriconazole plus an echinocandin may have survival benefits in hematology patients with invasive aspergillosis, there is no firm recommendation that combination therapy be administered first line but could be considered in select cases. A concerning observation is the emergence of a multidrug resistant species of *Aspergillus* causing breakthrough infections in patients with refractory leukemia and chronic GVHD after HSCT [65]. This species, *Aspergillus calidoustus*, exhibits high in vitro minimum inhibitory concentrations to multiple antifungal agents, including amphotericin B and voriconazole. In one case, serial combination antifungal therapy was ineffective and infection was universally fatal [66].

Mucormycosis, the disease caused by the *Mucorales* molds (e.g., *Rhizopus, Mucor, Rhizomucor*), is the second most common opportunistic mold infection seen in HSCT recipients. Risk factors include severe neutropenia, chronic GVHD, prolonged use of high dose corticosteroids, iron overload associated with transfusion dependence, iron chelator therapy and uncontrolled diabetes/diabetic ketoacidosis. Breakthrough infection may develop in HSCT recipients with GVHD who are receiving voriconazole prophylaxis or treatment. Mucormycosis usually manifests as an acute, rapidly progressive sino-orbital, pulmonary, cerebral or widely disseminated infection. In addition to antifungal therapy, management requires aggressive surgical debridement of devitalized tissue due to the propensity of these molds to invade blood vessels and cause necrosis. Patient outcomes may be improved when these strategies are combined with early diagnosis and reversal of underlying risk factors when feasible [67]. Amphotericin B, posaconazole and isavuconazole have activity against the *Mucorales* molds; however current guidelines recommend a lipid formulation of amphotericin B as first-line therapy of mucormycosis [42,67].

Posaconazole has not been studied as a primary treatment for mucormycosis but has shown some success when given as salvage therapy. In a retrospective study of 91 patients with mucormycosis (69 proven, 22 probable infections), overall success was 60% (14% complete and 42% partial response); a subgroup of 13 profoundly immunosuppressed patients who received at least one week of combination lipid formulation amphotericin B plus posaconazole had the same response rate as those who received posaconazole alone [68]. These results suggest that posaconazole may be considered a second-line agent or step down therapy following initial treatment with amphotericin B. Isavuconazole is approved for the treatment of mucormycosis based on a small, open label study of 37 patients with proven or probable infection [69]. Twenty-one patients received isavuconazole as primary treatment and 16 patients as salvage therapy; one-third of patients had undergone HSCT; and most patients had pneumonia with disseminated infection. Results showed a partial response to isavuconazole in 37 (11%) patients; 16 (43%) had stable disease and one (3%) had disease progression; all-cause mortality was 38%, but not unexpectedly, higher (46%) in patients with refractory mucormycosis. Based on the small sample size and non-randomized design of this study, as well as recent reports of breakthrough IFIs in patients treated with isavuconazole, more research will be needed to define the role of this agent in treating mucormycosis. The echinocandins do not have reliable in vitro activity against the *Mucorales* molds and should not be used as monotherapy; however, in one small, retrospective study, the addition of caspofungin to lipid formulation amphotericin B was superior to monotherapy [70]. Other retrospective reports have shown that combination therapy for mucormycosis offers no survival advantage compared to monotherapy, and at this time, is not recommended routinely [67].

*Fusarium* and *Scedosporium* are other rare molds that may cause opportunistic infection in HSCT recipients, particularly in those with chronic GVHD on immunosuppression [71]. Infection with these molds may mimic invasive aspergillosis, manifesting as pneumonia often with widely disseminated disease; *Fusarium* in particular may be associated with fungemia and necrotic skin lesions. These molds may have reduced in vitro susceptibility to amphotericin B, with some species having demonstrating multidrug resistance. Voriconazole is approved for treatment of refractory infections caused by *Fusarium* and *Scedosporium*; however, the mortality associated with these infections is high and outcome is dependent on control of the predisposing disease and immune status of the host.

##### Key Points

- The echinocandins (caspofungin, micafungin and anidulafungin) are first-line therapies against *Candida* infections; lipid formulations of amphotericin B are alternative therapies, especially if echinocandin resistance is suspected or documented; fluconazole can be used as step-down therapy once patients are clinically stable and antifungal susceptibility is confirmed.
- Yeasts other than *Candida*, such as *Trichosporon*, *Saccharomyces*, *Rhodotorula*, *Geotrichum* and *Pseudozyma*, are rare, and typically manifest as breakthrough fungemia in patients who are receiving antifungal prophylaxis; antifungal susceptibility patterns vary by isolate, but reduced susceptibility to echinocandins is common.
- Voriconazole is first-line therapy against *Aspergillus*; alternatives are lipid formulations of amphotericin B, isavuconazole, posaconazole, itraconazole and the echinocandins.
- Lipid formulations of amphotericin B are first-line therapies against Mucorales molds; alternatives are posaconazole and isavuconazole.
- Voriconazole can be used to treat *Fusarium* and *Scedosporium*; outcomes are dependent on control of the predisposing disease and host immune status.

## 4. Bacterial Infections

### 4.1. Prevention of Bacterial Infections during Neutropenia

Patients who undergo HSCT frequently experience prolonged neutropenia after myeloablative conditioning with chemotherapy, thereby increasing the risk of complications such as neutropenic fever and bacterial infections [72]. Antibacterial prophylaxis during periods of chemotherapy-related neutropenia has been associated with less febrile episodes and decreased frequency of microbiologically-documented infections [73]. Fluoroquinolones (FQs) have often been chosen as the antibiotic of choice for neutropenic prophylaxis due to associated reductions in Gram-negative bacteremia [73] and improved safety profile over some alternative antibiotics [74]. Most expert guidelines now recommend routine FQ prophylaxis for high-risk patients with expected profound and prolonged chemotherapy-induced neutropenia [23,30,75].

Despite widespread recommendations for use of FQs for prophylaxis, there have been new concerns regarding the safety profile of this antibiotic class. Since 2008, the US FDA has issued six safety alerts regarding potential adverse effects associated with reception of FQs, which include risk of tendinitis and tendon rupture; peripheral neuropathy; hypoglycemia; central nervous system effects; and most recently, aortic dissection and aneurysm. In addition, FQs have been reported to be associated with arrhythmia and QT prolongation [76], and neutropenic patients may be at particular risk due to concomitant reception of azole class antifungals for prophylaxis [23,30,75].

Another concern regarding the use of FQs for neutropenic prophylaxis has been the development of resistant bacterial infections. FQs have lesser activity against Gram-positive organisms, which remain significant pathogens in patients with malignancy due to the presence of lines and mucosal barrier injury [77]. Although certain FQs such as levofloxacin may be recommended for improved prophylactic activity against viridians group streptococcal (VGS) infection in patients with oral mucositis [75], HSCT recipients may develop breakthrough levofloxacin-resistant VGS bacteremia [78]. Cross-resistance between FQs in VGS infections can also occur; in one study of 48 neutropenic patients undergoing HSCT, levofloxacin-resistant VGS recovered from the oropharynx increased from 11% to 59% for all VGS isolates after only 8 days of gatifloxacin or moxifloxacin prophylaxis [79]. FQ-resistant Gram-negative infections have also been described [80,81]. In a single center study of patients undergoing HSCT, only 25% of extended-spectrum β-lactamase (ESBL)-producing Enterobacteriaceae recovered from perianal swabs were susceptible to levofloxacin prior to HSCT; after HSCT, 32% of patients colonized with ESBL Enterobacteriaceae developed subsequent bacteremia with the same ESBL Enterobacteriaceae, all of which were levofloxacin-resistant [80]. In another recent prospective multinational study of Gram-negative rod (GNR) bacteremia in patients undergoing autologous or allogeneic HSCT (auto-, allo-HSCT), half of all GNRs were FQ-resistant, and patients who were on FQs at time of bacteremia after allo-HSCT had 7-fold higher odds of breakthrough, FQ-resistant GNR infection [82].

Because of concerns for bacterial resistance and black box warnings associated with FQ use, alternative antibiotics for prophylaxis during neutropenia may be considered. Trimethoprim-sulfamethoxazole (TMP-SMZ) has been an attractive alternative to FQs for antibacterial prophylaxis given dual activity against infection with *Pneumocystis jiroveci.* A 2012 meta-analysis revealed no significant differences in all-cause or infection-related mortality, febrile episodes or bacteremia between patients who received TMP-SMZ or FQs for neutropenic prophylaxis [74]. However, use of TMP-SMZ was associated with more Gram-negative bacteremia and adverse effects when compared to FQs [30,74]. Although granulocytopenia has been the main adverse effect of concern when comparing TMP-SMZ to FQs for neutropenic prophylaxis [83,84], randomized controlled studies of TMP-SMX prophylaxis during neutropenia in patients with acute leukemia reveal no differences in myelosuppression compared to placebo [85] and suggest that TMP-SMZ may be a reasonable alternative to FQ prophylaxis in neutropenic patients undergoing HSCT.

Oral cephalosporin antibiotics have also been considered alternative choices for antibacterial neutropenic prophylaxis [86,87,88]. In a recent study of 142 patients who underwent allo-HSCT, patients who received cefpodoxime for pre-engraftment neutropenic prophylaxis had a similar incidence of neutropenic fever in comparison to those who received levofloxacin; in addition, there were no differences in the rate of multidrug-resistant infections within 100 days of HSCT [87]. Although in this study, *Pseudomonas aeruginosa* bacteremia only occurred in patients who received cefpodoxime, in another study of 120 patients with myelodysplastic syndrome, there was no difference in the incidence of *P. aeruginosa* infection between patients who received either cefpodoxime or cefdinir versus levofloxacin for neutropenic prophylaxis [86,87]. Retrospective studies such as these suggest that third-generation cephalosporins may be a reasonable alternative to FQs; however, future large-scale multicenter prospective studies should be conducted to determine true comparative clinical outcomes.

In 2012, a Cochrane meta-analysis of patients with chemotherapy-induced neutropenia concluded that antibiotic prophylaxis reduces all-cause mortality, with the most significant benefits having been seen in studies using FQs [74]. In contrast, in a more recent meta-analysis by the European Conference on Infections in Leukemia (ECIL), FQ prophylaxis during chemotherapy-induced neutropenia was not found to have mortality benefit, although reductions in bloodstream infections and febrile episodes were confirmed [89]. In patients who undergo HSCT, the risks of neutropenia on the development of fever and infection are well-known. However, with conflicting mortality data on FQ prophylaxis, as well as concerns over the development of breakthrough, resistant infections and the safety profile of the quinolone antibiotic class, future prospective studies with antibiotic agents such as third-generation cephalosporins may provide safer alternatives in high-risk neutropenic patients.

#### Key Points

- Most guidelines recommend antibacterial prophylaxis with fluoroquinolones for patients with profound and prolonged chemotherapy-induced neutropenia.
- However, fluoroquinolones have many potential side-effects, which include tendinitis and tendon rupture, peripheral neuropathy, hypoglycemia, central nervous system effects, QT prolongation and aortic dissection and aneurysm; the development of resistant bacterial infections is also a problem.
- Alternatives to fluoroquinolones are trimethoprim-sulfamethoxazole or oral third-generation cephalosporins, such as cefpodoxime or cefdinir; large multicenter prospective studies are needed to determine their comparative effectiveness.

### 4.2. New Therapeutics in Resistant Gram-Negative Infections

The incidence of infections with multi-drug resistant organisms (MDROs) is increasing globally, and HSCT recipients are especially vulnerable, given their weaker immune systems [90,91]. A recent study of HSCT recipients found that 40.5% of patients are intestinally colonized with MDROs and 20% develop bloodstream infections with resistant organisms [92]. Infection with resistant organisms among HSCT recipients is associated with increased mortality [90,93]. Given the vulnerability of these immunocompromised hosts to MDRO infections, preventing acquisition of these resistant organisms, as well as treating them adequately when they do occur, is very important.

The IDSA recommends that all hospitals have antibiotic stewardship programs to aid judicious antimicrobial use and to curtail the rising incidence of MDROs. Studies have shown that stewardship strategies such as hospital-based antibiotic restriction and cycling have improved bacterial susceptibility profiles in units under investigation [94]. Of interest to the HSCT community, antibiotic cycling for treatment of febrile neutropenia in a hematologic malignancy ward (i.e., patients already receiving fluoroquinolone prophylaxis) has demonstrated reduced recovery of MDROs without changing the rate of treatment failure [95].

While preventing colonization and infection by resistant pathogens with the best stewardship and infection control strategies is important, targeted and effective treatment of MDRO infections is also necessary. There are several new antibiotics or antibiotic combinations which may be of use in the treatment of resistant Gram-negative infections.

Eravacycline (Xerava^TM^) is a tetracycline-class antimicrobial that has activity against Gram-positive (including MRSA), Gram-negative and anaerobic organisms [96]. It has activity against extended-spectrum beta-lactamase (ESBL) and carbapenemase producing organisms. *Pseudomonas* and *Burkholderia* species are gaps in coverage. Suggested dosing for eravacycline in clinical use is 1 mg/kg IV every 12 h, with dose escalation to 1.5 mg/kg every 12 h if a strong CYP3A inducer is given concomitantly [97]. No renal adjustments are suggested. For patients with severe hepatic impairment, a dose reduction to 1 mg/kg every 24 h is recommended. Gastrointestinal secondary effects were common in the clinical trials; however, eravacycline should also be presumed to have other tetracycline-related class-specific side-effects (photosensitivity, tooth discoloration) as well. To preserve this novel drug’s activity, it would be prudent to restrict use to treatment for intra-abdominal infections with confirmed or probable drug resistance (ESBL) intolerant of carbapenems. Notably, the steady state volume of distribution for this agent is elevated (beyond a typical plasma distribution) at 3.3L/kg, which may limit its use with bloodstream infections [97].

Plazomicin (Zemdri^TM^) is an aminoglycoside-class antibiotic that has structural modifications that can overcome bacterial resistance to other aminoglycoside antibiotics [98]. It has activity against aerobic Gram-negative bacteria, and has a low volume of distribution. Suggested dosing is 15 mg/kg daily. Side effects include nephrotoxicity, which is more common in patients with pre-existing renal insufficiency. Dose-reduction is suggested for CrCl ≤ 60 mL/min, and like other aminoglycosides dosing is modified for extremes of weight. Use of plazomicin in HSCT recipients will likely be in patients with multidrug-resistant Gram-negative bacteria (CRE, ESBL) resistant to other aminoglycosides, such as amikacin, gentamicin or tobramycin. The relative rarity of amikacin-resistant aerobic Gram-negative bacteria, however, will lead to very restricted use.

Meropenem-vaborbactam (Vabomere^TM^) is a novel drug combination that pairs meropenem with a newly developed beta-lactamase inhibitor. Vaborbactam, while not having anti-bacterial effects of its own, binds and inhibits the serine protease portion of carbapenemase enzymes such as *Klebsiella pneumoniae* carbapenemase (KPC) via a boronic acid moiety, and prevents their cleavage of beta-lactam rings [99]. It has activity against certain carbapenemase-producing Gram-negative bacteria. It is dosed at 4 g every 8 h. If CrCl is less than 50 mL/min, reduced dosing is advised. Significant interactions with valproic acid or derivatives and probenecid have been noted. As meropenem-vaborbactam is a beta-lactam-based antibiotic, patient-reported reactions to structurally similar drugs should be considered prior to prescription.

Ceftazidime-avibactam (Avycaz^TM^) pairs ceftazidime, a third-generation cephalosporin with anti-pseudomonal activity, with avibactam, a new beta-lactamase inhibitor. Avibactam reversibly acylates beta-lactamase enzymes—that prevents their destruction of antibiotics with beta-lactam groups—while also binding to some penicillin-binding-proteins in in-vitro studies [100]. This combination has demonstrated activity against *Enterobacteriaceae*, including those producing penicillinases and cephalosporinases [100]. The typical starting dose is 2.5 g q8 h IV. CrCl less than 50 mL/min or ESRD patients require dosage adjustments. Use in HSCT would likely be in patients with resistant *Pseudomonas aeruginosa* infections and patients with CRE.

Ceftolozane-tazobactam (Zerbaxa^TM^) pairs a novel anti-pseudomonas cephalosporin with the beta-lactamase inhibitor tazobactam [101]. Ceftolozane-tazobactam’s spectrum of activity also includes ESBL producing organisms. Dosing is suggested at 1.5g every eight hours with adjustment made for CrCl ≤ 50 mL/min [102]. Hypersensitivity to structurally related antimicrobials should be taken into consideration prior to prescribing. Niche use of this novel cephalosporin and beta-lactamase inhibitor combination will likely be infections with ESBL *Enterobacteriaceae* and MDR *Pseudomonas.*

#### Key Points

- Hospitals should have antimicrobial stewardship programs to aid judicious antibiotic use and prevent the emergence of multidrug-resistant organisms; novel antibiotics with activity against resistant Gram-negative organisms should have protected status and should be used only when absolutely necessary.
- Eravacycline is a tetracycline-class antimicrobial with activity against Gram-positive (including MRSA), Gram-negative (including ESBL and carbapenemase producing organisms, but not *Pseudomonas* or *Burkholderia*) and anaerobic bacteria; it has a large volume of distribution which will limit its use with bloodstream infections.
- Plazomicin is an aminoglycoside-class antibiotic with activity against aerobic Gram-negative bacteria that are resistant to other aminoglycoside antibiotics; therapeutic drug monitoring is required to decrease toxicity.
- Meropenem-vaborbactam has activity against carbapenamase producing organisms; vaborbactam, while not having anti-bacterial effects of its own, binds and inhibits carbapenemase enzymes such as KPC, thereby preventing cleavage of beta-lactam rings.
- Ceftazidime-avibactam and ceftolozane-tazobactam have activity against penicillinase, ESBL and cephalosporinase-producing Gram-negative bacteria (including *Pseudomonas* and *Burkholderia*); it has reduced activity against anaerobes.

## 5. Clostridium Difficile Infection

### 5.1. Updates on Prevention of Clostridium Difficile Infection

Infection with *Clostridium difficile* in patients who undergo HSCT is well-described [103,104].

In a recent prospective study of allogeneic HSCT (allo-HSCT) recipients in the Organ Transplant Infection Project, *C. difficile* was the most common bacterial infection with 33% of patients having at least one episode and 26% of patients having recurrence [105]. The incidence of *C. difficile* infection (CDI) may be up to 9-fold higher in HSCT recipients than that of the general population [106], with most infections occurring early in the post-transplant period [105,107,108]. Risk factors for CDI in HSCT recipients include reception of chemotherapy prior to conditioning, exposure to high-risk antibiotics (e.g., fluoroquinolones and broad-spectrum β-lactam antibiotics) and mucositis [103,107,109,110]. The association between CDI and GVHD is complex; CDI is associated with gastrointestinal (GI) GVHD, which in turn predisposes to recurrent CDI [103,111].

Recent studies on CDI prophylaxis are worth mentioning. In a retrospective single center study of 145 patients admitted for allo-HSCT, reception of prophylactic oral vancomycin 125 mg twice daily throughout their hospital stay was associated with no cases of CDI; whereas 20% of patients developed CDI in the non-prophylaxis group (0/90 vs. 11/55, respectively; *p* < 0.001) [112]. Antibiotic exposure within 30 days after allo-HSCT and risk of CDI relapse was similar between the two groups, and the reception of oral vancomycin was not associated with increased risk for GVHD, despite theoretical concerns of microbiome disruption [112]. The use of once daily prophylactic fidaxomicin 200 mg in both auto- and allo-HSCT recipients was studied in a randomized placebo-controlled double-blinded trial of 611 patients from 42 medical centers in North America; in patients who received fidaxomicin prophylaxis from conditioning through neutrophil engraftment or completion of fluoroquinolone prophylaxis, there was significantly less confirmed *C. difficile*-associated diarrhea than for those who received the placebo [113]. Overall drug-related adverse events were higher in the placebo group in comparison to the fidaxomicin group (20% vs. 15%, respectively), and only 4/300 patients in the fidaxomcin group and 2/300 patients in the placebo group were deemed to have drug-related, serious adverse effects [113]. These studies suggest that CDI prophylaxis may be beneficial in the early-post HSCT period, although it is unclear if other HSCT recipients with high-risk features (i.e., mucosal barrier injury, reception of antibiotics) will also benefit from prophylaxis.

#### Key Points

- Infection with *C. difficile* continues to be a major cause of morbidity in HSCT recipients.
- Prophylactic oral vancomycin and fidaxomicin may prevent *C. difficile* associated diarrhea in certain subsets of HSCT patients.

### 5.2. Updates in Treatment of Clostridium Difficile Infection

In 2017, the Infectious Diseases Society of America (IDSA) and Society for Healthcare Epidemiology of America (SHEA) updated the existing 2010 clinical practice guidelines for CDI [114]. Major changes to the previous guidelines included the addition of oral vancomycin or fidaxomicin for first-line treatment (rather than oral metronidazole), the option of prolonged vancomycin taper for a first recurrence (rather than second recurrence only) and the consideration of fecal microbiota transplantation (FMT) after ≥3 recurrences [114,115,116]. Several randomized controlled trials and meta-analyses have demonstrated the improved efficacy of oral vancomycin over oral metronidazole for CDI treatment, and the likely benefit of fidaxomicin over oral vancomycin at preventing recurrence [117,118,119,120]. In a 2013 meta-analysis, cure rates for CDI with FMT were found to be near 90% [121]; however, this primarily included observational studies, and a more recent meta-analysis of randomized trials suggested that cure rates may be closer to 67%, with variability in outcomes dependent on route of FMT administration [122]. Bezlotuxumab, a monoclonal antibody against *C. difficile* toxin B was approved by the FDA in October 2016 for treatment and prevention of recurrent CDI in adults [123]; however, this drug has not yet been recommended for routine use in the current guidelines.

Diagnostic and treatment recommendations with standard antimicrobials for CDI in HSCT recipients are expected to be the same as the general population; however, a few unique features and the concern of FMT safety in this population are worth mentioning. Asymptomatic *C. difficile* colonization in HSCT recipients has been noted to be as high as 29%; compare that to the up to 15% in healthy adults [124,125]; this high rate of colonization coupled with the high incidence of diarrhea overall (i.e., due to mucosal barrier injury with cytotoxic therapy, GVHD other infections such as CMV) may present diagnostic challenges in identifying true CDI [126,127]. Damage to the gut barrier may also have therapeutic implications; for instance, severe GI GVHD has been associated with systemic absorption to supra-therapeutic levels of oral vancomycin for concomitant CDI treatment, with potential effects on renal function [128]. Lastly, FMT in HSCT recipients is controversial, although potential uses may be wide-ranging to include restoration of disrupted microbiome (i.e., higher mortality associated with lower intestinal diversity [129]) and treatment of acute GVHD and recurrent CDI [130]. Several case reports and series have now demonstrated efficacy and safety of FMT for recurrent CDI treatment in both autologous (auto-) and allo-HSCT recipients, which was administered by a variety of methods (capsules, enemas, naso-jejunal tubes, push enteroscopy/colonoscopy) [131,132,133,134,135]. No donor-derived infections were reported, with some studies performing extensive behavioral and microbiologic (serum, stool) screening of donors prior to FMT. [132,133] Findings such as these suggest that FMT may be considered for HSCT recipients in the future, especially in the setting of recurrent CDI where disease persistence, severity and relapse may be affected by factors such as continued immunosuppression and use of broad-spectrum antibiotics [110,133,135].

#### Key Points

- Recent treatment guidelines recommend the use of oral vancomycin or fidaxomicin as the first line for the treatment of CDI in the general population.
- The use of FMT in HSCT recipients may be a safe method for curing recurrent CDI when performed with adequate donor screening and recipient evaluation.
- The utility of bezlotuxumab has not yet been assessed in HSCT patients, but may hold some promise.

### 5.3. Epstein Barr Virus

Epstein Barr virus (EBV) is a double-stranded DNA virus that is a member of the Herpesviridae family [136]. Primary EBV infection of epithelial cells and B-lymphocytes usually occurs at a young age, followed by life-long latency in a subset of B-lymphocytes. In immunocompetent hosts, active immune surveillance and functional T-lymphocytes control latently-infected B-lymphocytes [136]. However, immune dysfunction can lead to unchecked EBV-mediated latent gene expression of survival genes, resulting in unchecked B-lymphocyte proliferation and monoclonal expansion with malignant transformation [136].

The clinical manifestations of EBV infection in immunocompetent hosts can range from asymptomatic infection to primary infectious mononucleosis or EBV-associated malignancies, such as nasopharyngeal carcinoma or Burkitt’s lymphoma. In HSCT, the most feared complication of uncontrolled EBV-mediated B-lymphocyte clonal proliferation is post-transplant lymphoproliferative disorder (PTLD) [136]. PTLD is a heterogeneous group of complex lymphoproliferative disorders that reflect step-wise acquisition of B-lymphocyte mutations that culminate in malignancy [136,137,138]. Risk factors for EBV-associated PTLD are primarily related to immunosuppression and T-lymphocyte dysfunction, which may include T-cell depletion of the graft [139]. Other risk factors include EBV serodiscordance; seronegative recipients who receive HSCT from seropositive donors (D+/R-) are at greatest risk of developing PTLD with an odds ratio of up to 13.6, possibly due to uncontrolled EBV-infected lymphocyte proliferation in an EBV-naïve host [139,140,141].

The mainstay of EBV-associated PTLD management is reduction in immunosuppression. Other therapy options include rituximab, donor lymphocyte infusion, EBV-specific cytotoxic T-cells and chemotherapy [137]. After HSCT, some centers monitor for EBV viremia and reduce immunosuppression or consider rituximab for B-cell depletion pre-emptively; this strategy is recommended by the Sixth European Conference on Infections in Leukemia (ECIL-6), and entails performing weekly quantitative EBV PCR tests from whole blood, plasma or serum from the first through fourth month post-transplant [138]. However, the optimal EBV viremia threshold for which pre-emptive therapy should be instituted is unknown, and leads to significant practice variability [142,143,144]. Suggested EBV viral load cut-offs for rituximab range from 100 to 10,000 copies/mL [140,142,145,146]. In a retrospective study of 554 patients who underwent anti-thymocyte globulin-conditioned myeloablative HSCT, there was no difference in mortality between patients who received pre-emptive therapy based on EBV surveillance and those who were treated for biopsy-proven PTLD without EBV viral load monitoring; however, the viral load threshold for the pre-emptive group was relatively high at >40,000 DNA copies/mL [141]. In another retrospective study of 332 HCT recipients, a survival benefit was found for patients with EBV viral load ≥50,000 copies/mL who received rituximab [147]. Additional markers such as T-cell reconstitution may be an important adjunct to EBV viremia surveillance when weighing the risks and benefits of pre-emptive therapy [148].

Antiviral agents are ineffective in the prevention or treatment of EBV-associated PTLD and are not recommended for EBV prophylaxis in HSCT recipients [138,139,149]. In vitro anti-viral agents, such as foscarnet, acyclovir and valganciclovir/ganciclovir, inhibit EBV DNA polymerase and stop viral replication [139,150]. However, no lytic replication occurs in B-lymphocytes latently infected with EBV, rendering antiviral agents ineffective [149,151] Small case series and phase 1 and 2 trials suggest that certain patients with PTLD and other lymphoid malignancies who express some lytic genes may be more susceptible to foscarnet, and that the addition of arginine butyrate may increase susceptibility to ganciclovir by inducing thymidine kinase expression; however, more studies are needed to validate these findings [152,153]. Current clinical trials related to anti-viral therapy focus on human leukocyte antigen-matched virus-specific T-cell therapy and do not include standard antiviral agents (ClinicalTrials.gov: NCT03475212).

#### Key Points

- In HSCT, EBV-mediated B-lymphocyte proliferation and monoclonal expansion with malignant transformation can culminate in PTLD.
- Reduction in immunosuppression is the mainstay of EBV-associated PTLD management. Other therapy options include rituximab, donor lymphocyte infusion, EBV-specific cytotoxic T-cells and chemotherapy.
- Monitoring for EBV viremia after HSCT followed by reduction of immunosuppression or rituximab has been recommended; however, studies are conflicting on whether it impacts mortality.
- Antiviral agents are ineffective in the prevention or treatment of EBV-associated PTLD.

### 5.4. Human Herpes Virus-6 (HHV-6)

Human herpes virus-6 (HHV-6) is a ubiquitous beta-herpesvirus that can cause opportunistic infection and disease in stem cell transplant recipients. It is characterized by an encapsidated linear double-stranded DNA genome, latency after primary infection, periodic reactivation and direct person-to-person transmission [154]. HHV-6 is divided into two genetically and phenotypically distinct variants referred to as HHV-6A and HHV-6B [155]. It can persist indefinitely in the host through episomes within cell nuclei [156], or chromosomal integration [157,158], a unique feature which allows person-to-offspring transmission through germinal cells [158].

The prevalence of HHV-6 infection as determined by seropositivity in the general population is 90% [159]. Current serologic techniques cannot differentiate between HHV-6A and HHV-6B. The vast majority of infections are due to HHV-6B. Reactivation in immunocompromised hosts is common, and occurs in 30 to 70 percent of patients undergoing allogeneic SCT [160,161,162,163,164]. Disease, however, is uncommon, with encephalitis as the most clearly recognized clinical manifestation. Risk factors for reactivation include umbilical cord SCT with rates up to 90 percent [164,165,166,167,168]; unrelated or mismatched donors [163,164,165,166]; and myeloablative conditioning [164].

HHV-6B encephalitis uncommonly occurs after reactivation in allogeneic SCT recipients and results in considerable morbidity [169,170,171]. It usually presents between two and six weeks after transplantation, and is characterized by confusion, anterograde amnesia and occasional seizures [171]. Other symptoms include personality changes, irritability and apathy [172]. The CSF cell count and protein concentration are typically normal or slightly elevated [171,172]. MRI shows hyperintensities in the medial temporal lobes, particularly the amygdala and hippocampus [170,173], and EEG can show epileptiform activity or diffuse slowing [171,172].

Diagnosis is made by direct detection of the virus, typically by PCR. PCR can be performed on CSF, peripheral blood samples or tissue. Detection by PCR can reflect active or latent infection, depending on the clinical setting and the specimen tested. Quantitative PCR should be performed when possible to determine trends over time [174,175]. There are no cut-offs that reliably discriminate between disease and latency, so these tests must be interpreted within the clinical context. Acute encephalopathy coupled with detection of HHV-6 in CSF and exclusion of other causes is diagnostic of HHV-6B encephalitis [164,171,176,177]. Detection of HHV-6 in peripheral blood can be helpful in establishing HHV-6 reactivation, but is not specific for encephalitis. Inherited chromosomal integration of HHV-6 in donor or recipient cells can cause high viral loads in the blood or CSF by PCR in the absence of active infection, and can confound diagnosis [178,179,180]. Chromosomally integrated HHV-6 should be considered in persons with high HHV-6 viral loads with no encephalitis signs or symptoms, and those with viremia that does not decline with anti-viral therapy.

Treatment of HHV-6B encephalitis involves antiviral therapy and anticonvulsant therapy when seizures are present. Foscarnet, ganciclovir and cidofovir have activity against HHV-6B [159], but foscarnet and ganciclovir are considered first-line given that cidofovir is highly nephrotoxic. Full-dose foscarnet or ganciclovir are more effective than lower dose therapy [181]. For patients with normal renal function, foscarnet 60 mg/kg IV every 8 h or 90 mg/kg IV every 12 h or ganciclovir 5 mg/kg IV every 12 h should be given [182,183,184]. Both antiviral agents have significant toxicities. Foscarnet can cause electrolyte depletion, which can lower the seizure threshold, as well as nephrotoxicity. Ganciclovir can cause bone marrow suppression. Therapy should be given for at least 21 days, but could be prolonged in patients with poor clinical response and/or persistently detectable virus in blood or CSF. Blood HHV-6B viral load should be checked weekly during therapy until it becomes undetectable. Repeat CSF HHV-6B viral load can be done in patients who do not improve despite appropriate antiviral therapy. Levetiracetam can be used as an anti-seizure agent, since it does not affect cytochrome P450 and will not interact with GVHD or antifungal prophylaxis or treatment. Prophylactic foscarnet or ganciclovir has not been shown to prevent HHV-6B encephalitis [185].

Outcomes of HHV-6B encephalitis vary widely, with some patients recovering full neurological function and other patients being left with residual neurologic deficits [169,171].

#### Key Points

- The majority of infections are due to HHV-6B. Reactivation after HSCT is common, but encephalitis is uncommon.
- Encephalitis presents between two and six weeks after HSCT, and is characterized by confusion, anterograde amnesia and seizures. MRI shows hyperintensities in the medial temporal lobes.
- Diagnosis is made by direct virus detection by PCR. Acute encephalopathy coupled with detection of HHV-6 in CSF and exclusion of other causes is diagnostic of HHV-6B encephalitis. Inherited chromosomal integration of HHV-6 in donor or recipient cells can cause high viral loads in the blood or CSF by PCR in the absence of active infection, and can confound diagnosis.
- Treatment of HHV-6B encephalitis includes antiviral therapy with foscarnet or ganciclovir, and anticonvulsant therapy when seizures are present.

### 5.5. Adenovirus

Adenoviruses are double-stranded DNA viruses that are important pathogens in HSCT recipients. Adenoviral disease may result from de novo infection or reactivation of persistent endogenous virus, with the latter being the predominant cause in HSCT recipients [186,187]. Adenovirus infections have an incidence of up to 21%, with frequency of invasive disease as high as 8% to 26% and an associated mortality of up to 50% in HSCT recipients [188,189,190,191,192,193,194]. Pneumonia and disseminated disease are associated with a higher mortality rate [188]. Adenovirus infections are more common in allogeneic than autologous HSCT recipients, and risk factors include a haploidentical or unrelated cord blood donor; graft-versus-host disease; reception of multiple immunosuppressive agents, including anti-T cell antibodies (e.g., ATG, alemtuzumab); and severe lymphopenia (<200 cells/μL peripheral blood specimen) [183,188,195,196,197].

Adenovirus may cause an array of clinical syndromes following HSCT, including asymptomatic viremia, encephalitis, conjunctivitis, upper respiratory infection, pneumonia, myocarditis, enteritis, hepatitis, hemorrhagic cystitis, nephritis, colitis and disseminated disease [188,195]. Disseminated disease may manifest as a combination of these syndromes in the presence of two or more positive PCR assays obtained from peripheral blood and additional sites (e.g., cerebrospinal fluid, bronchoalveolar lavage fluid, respiratory secretions or urine) [186,188].

Conventional viral culture had previously been the standard diagnostic test. Newer diagnostic techniques with reasonable sensitivity and specificity and improved time to diagnosis include viral PCR, shell vial culture and immunochromatographic techniques [196,198,199]. PCR is more sensitive and specific than culture or antigen detection and is equally sensitive for all adenovirus serotypes. Sensitivity for PCR has been reported at 94% [196,199,200]. A pre-emptive approach is recommended for patients at highest risk of developing adenoviral infection; guidelines recommend weekly monitoring of serum adenovirus PCR for either the first six months after HSCT or the duration of severe immunosuppression/lymphopenia [183,186,201]. In the absence of definitive data, no recommendations are made regarding a critical value for viremia to initiate treatment [183]. However, sustained or high-level viremia detection by PCR-based analysis may indicate underlying adenovirus disease and has been associated with fatal disease [186,201]. A ten-fold increase in viral load has been found to precede clinical signs of adenovirus disease by a median of three weeks [186]. Therefore, pre-emptive antiviral therapy with cidofovir or ribavirin may be utilized for selected high-risk patients [183].

Treatment of proven or probable adenovirus disease involves a combination of reduction of immunosuppression (rapid tapering or withdrawal) and antiviral therapy [183,196]. No randomized, placebo-controlled trials of antiviral therapy for adenoviral infection have been performed, and few treatment options are available. Cidofovir has been successfully used to treat serious adenovirus infection in HSCT recipients [202]. A retrospective analysis of forty-five patients treated with cidofovir found a success rate of 69%; however, other studies have reported as high as 100% mortality in subsets of patient with adenovirus pneumonia while receiving cidofovir [203,204]. Cidofovir may be dosed as either 5 mg/kg weekly, or alternatively, 1 mg/kg three times per week, noting that the lowered dose may cause lesser renal toxicity [196,205]. Renal toxicity attributed to proximal tubular dysfunction has been cited as a frequent adverse event that may limit therapy. Oral probenecid and adequate hydration is recommended concurrently with cidofovir administration [203]. Probenecid’s mechanism of renal protection is unknown. It has been postulated that concomitant high-dose probenecid may decrease renal clearance of cidofovir by blocking active tubular secretion of the drug. Probenecid should be administered 2 g orally three hours prior to cidofovir infusion, followed by 1 g orally at two and eight hours after completing cidofovir infusion.

Brincidofovir is an orally bioavailable and less nephrotoxic lipid ester of cidofovir that has efficacy against adenovirus [206,207]. Twice weekly brincidofovir dosed at 2 mg/kg has been successful in treating viremia and depicting a lower but non-significant mortality rate [207,208,209]. Advantages of brincidofovir over other antiviral agents include central nervous system penetration and absence of nephrotoxicity or myelosuppression [207]. Diarrhea and increased risk of acute graft-versus-host disease are important adverse events [208]. While brincidofovir remains an investigational agent, it is available for patients with severe adenovirus infections as part of the expanded access program.

Recent evidence has highlighted the role of adenovirus-specific cytotoxic T lymphocytes (CTL) in the treatment of adenoviral disease. CTL therapy provides adoptive transfer of immunity to patients with adenovirus infection by providing sustained in vivo expansion of adenovirus-specific T-cells [210,211]. CTL may be harvested via leukapheresis from original donors of HSCT or a third-party haploidentical donor. While viremia clearance has been noted in up to 91% of patients, GVHD is an important side-effect of this therapy. CTL therapy is experimental and can only be utilized in clinical trials at specialized centers [210,211]. Ongoing clinical trials registered at clinicaltrials.gov include NCT00590083 and NCT03266627. In addition to routine hand hygiene, guidelines recommend droplet plus contact precautions for at least the duration of illness to prevent transmission between hospitalized patients [183].

#### Key Points

- Adenovirus can cause many clinical syndromes in HSCT patients, which include respiratory infection, encephalitis, nephritis, colitis and disseminated disease.
- Diagnosis is commonly made by PCR-based virus detection.
- Treatment involves a combination of reduction of immunosuppression and antiviral therapy. Options for antiviral therapy include cidofovir and brincidofovir.

### 5.6. BK Virus

BK is a double-stranded DNA-based polyoma virus with tropism for urothelium and renal tubular cells. It is common in the general population but typically only causes disease in severely immunocompromised hosts [212]. In HSCT, BK virus has a variety of manifestations, including hemorrhagic cystitis, and less commonly, strictures of urothelium and renal dysfunction [213,214,215]. While the conditioning regimen for HSCT may cause a hemorrhagic cystitis in the pre-engraftment period, hemorrhagic cystitis in the early post-engraftment period often relates to BK virus [216]. This temporality is thought to be due to immune reconstitution causing an inflammatory response to replicating BK virus in already damaged urothelium [216]. Another agent on the differential for post-engraftment hemorrhagic cystitis in this population is adenovirus. While rare, cases of encephalitis and pneumonitis secondary to BK virus in HSCT have been described [217,218,219].

When BK virus is suspected based on clinical findings (irritative voiding symptoms without bacterial isolation, urinary outflow obstruction, hematuria), the diagnosis may be established by PCR testing for viral DNA in the urine and blood. The median time from transplant to detection of viremia was 68 days in one study, and viruria typically precedes viremia [220]. Positive pre-transplant BK virus serology has been associated with development of viruria following conditioning, and rapid increases in viral load have been shown to be associated with hemorrhagic cystitis [221]. Other risk factors for development of BK virus associated hemorrhagic cystitis are allogeneic as opposed to autologous SCT; a fully myeloablative conditioning regimen; and an unrelated marrow donor [213,222,223].

BK virus replication may be encountered via PCR testing without evidence of infection or symptoms. Apart from monitoring viral load, the optimal management of asymptomatic BK viremia or viruria is unclear. A small study noted that universal prophylaxis of HSCT patients with ciprofloxacin appeared to diminish rates of BK associated hemorrhagic cystitis; however, these results have not been replicated [224].

Management of BK virus hemorrhagic cystitis primarily consists of supportive care to maintain patient comfort, renal function and hemodynamics. [225]. Use of intravenous or intravesicular cidofovir has been reported in small case series [226,227]. However, it is unclear whether clearance of BK virus is related to cidofovir use or immune recovery. Recent in vitro studies show that artesunate has activity against BK virus; however, no clinical trials have been done. [228,229,230]. Donor lymphocyte infusion has been used for refractory BK-associated hemorrhagic cystitis [231]; however, further studies are needed.

#### Key Points

- BK virus has a tropism for urothelium and renal tubular cells. In HSCT, BK virus can cause hemorrhagic cystitis, and less commonly, strictures of urothelium and renal dysfunction.
- Clinical manifestations include irritative voiding symptoms, urinary outflow obstruction and hematuria.
- Diagnosis is established by PCR testing for viral DNA in urine and blood.
- Treatment is largely supportive, and includes ensuring patient comfort, renal function and hemodynamics. Reduction of immunosuppression is also important. Use of intravenous or intravesicular cidofovir has been reported.
